# Ongoing viral replication and production of infectious virus in patients with chronic hepatitis B virus suppressed below the limit of quantitation on long-term nucleos(t)ide therapy

**DOI:** 10.1371/journal.pone.0262516

**Published:** 2022-04-01

**Authors:** Dara L Burdette, Scott Lazerwith, Jenny Yang, Henry L. Y. Chan, William E. Delaney IV, Simon P. Fletcher, Tomas Cihlar, Becket Feierbach

**Affiliations:** 1 Discovery Virology, Gilead Sciences, Foster City, CA, United States of America; 2 Medicinal Chemistry, Gilead Sciences, Foster City, CA, United States of America; 3 Clinical Research, Gilead Sciences, Foster City, CA, United States of America; 4 The Chinese University of Hong Kong, Hong Kong SAR, China; 5 Clinical Virology, Gilead Sciences, Foster City, CA, United States of America; University of Pittsburgh School of Medicine, UNITED STATES

## Abstract

Nucleos(t)ide analogs are standard-of-care for the treatment of chronic hepatitis B and can effectively reduce hepatitis B virus (HBV) replication but rarely leads to cure. Nucleos(t)ide analogs do not directly eliminate the viral episome, therefore treatment cessation typically leads to rapid viral rebound. While treatment is effective, HBV DNA is still detectable (although not quantifiable) in the periphery of the majority of nucleos(t)ide analog treated HBV patients, even after prolonged treatment. Addressing whether the detectable HBV DNA represents infectious virus is a key unknown and has important implications for the development of a curative treatment for HBV. The minimum HBV genome equivalents required to establish infection in human liver chimeric mice was determined by titration of HBV patient sera and the infectivity in chimeric mice of serum from patients (n = 7) suppressed to the limit of detection on nucleos(t)ide analog therapy was evaluated. A minimum of 5 HBV genome equivalents were required to establish infection in the chimeric mice, confirming this model has sufficient sensitivity to determine whether serum from virally suppressed patients contains infectious virus. Strikingly, serum from 75% (n = 3 out of 4) of nucleos(t)ide-treated HBV patients with DNA that was detectable, but below the lower limit of quantitation, also established infection in the chimeric mice. These results demonstrate that infectious virus is still present in some HBV patients on suppressive nucleos(t)ide therapy. This residual virus may support viral persistence via continuous infection and explain the ongoing risk for HBV-related complications despite long-term suppression on therapy. Thus, additional treatment intensification may facilitate HBV cure.

## Introduction

Hepatitis B is a global epidemic and a major cause of liver disease, including cirrhosis and liver cancer. The World Health Organization estimates approximately 257 million individuals have chronic hepatitis B (CHB) worldwide resulting in an estimated 887,000 related deaths in 2015 [1]. Widespread vaccination programs have successfully reduced HBV global prevalence preventing newly acquired infections but are ineffective at treating CHB patients that are already chronically infected [2,3]. Current direct-acting antiviral treatments for CHB target the HBV reverse transcriptase/polymerase and while viral suppression can be achieved in the majority of patients on long-term nucleos(t)ide analog (NA) therapy, treatment rarely results in functional cure (i.e. sustained HBsAg loss) and patients continue to be at-risk for development of hepatocellular carcinoma (HCC) [4–11]. Ideally patients on long-term NA therapy achieve HBV DNA suppression, which is defined as ≤162 copies per milliliter (mL). Serum from these suppressed patients can be further stratified into two groups defined by the limits of the polymerase chain reaction (PCR) assays used to measure HBV DNA. HBV DNA serum levels below the lower limit of quantitation (LLOQ) of 162 copies per mL that can still be detected, but not accurately quantified, is defined as target detected (TD). Conversely, HBV DNA serum levels below the LLOQ that are also below the lower limit of detection (LLOD) and not detected by PCR-based assays are referred to as target not detected (TND). We previously reported that after 240 weeks of treatment with the NAs tenofovir disoproxil fumarate (TDF) or TDF and emtricitabine (FTC), HBV DNA levels in the serum of the majority of CHB patients reached TD status [12]. Moreover, Human Immunodeficiency Virus (HIV)-HBV co-infected patients on long-term TDF therapy still have very low but detectable levels of intrahepatic covalently closed circular DNA (cccDNA) [13]. Taken together, these data suggest that viral suppression is not complete with the current NA therapies. However, it is not known whether serum below the LLOQ contains infectious virus. If there remain significant levels of circulating infectious virus in patients on NA treatment, additional therapies for viral suppression would be needed to achieve full suppression that might facilitate HBV cure.

Mouse models for the study of HBV include human chimeric mice (uPA-SCID and FAH), and models where HBV transcription is driven by adenoviral transduction or replicons delivered by hydrodynamic injection [14]. Non-human primates have been used to study HBV [15–17]. However, chimpanzees are now heavily restricted in biomedical research. Studies have demonstrated that sera from patients with an active viral infection (high viral load) diluted to contain low amounts of virus can lead to chronic infection in multiple animal models including a study demonstrating an inoculum of serum diluted to 1 GE of HBV is capable of infecting chimpanzees [18]. Although immune compromised, human chimeric mice are the only mouse models that can be naturally infected with HBV resulting in sustained infection with viral spread. The uPA-SCID and FAH models have been used extensively to study viral infection [19–22], however there have been no studies to determine if serum from patients suppressed on NA therapy is still infectious. Here, we use the uPA/SCID human chimeric mouse model to assess the infectivity of serum from patients prior to and after long-term NA therapy. Specifically, we determine if serum from patients with HBV DNA levels below LLOQ but still TD contain infectious virus capable of infecting new hepatocytes.

## Methods

### Patient samples

Nine patients chronically infected with HBV genotype B or C were selected from a phase 3 clinical study (GS-US-203-0101) that evaluated TDF ± FTC for 208 weeks for the treatment of CHB [12,23] (Table 1). Patients supplied written informed consent prior to study procedures.

**Table 1:** Viral kinetics of the patients selected for this study following administration of TDF or TDF/FTC. Timepoints indicate time on NA treatment at which serum was sampled. HBV DNA was measured by Roche TaqMan COBAS at the indicated timepoints (copies/mL). Shaded boxes indicate HBV DNA levels reached LLOQ and are ≤ 162 copies/mL.

	Viral Load (Copies/mL)								
Patient Number	1	2	3	4	5	6	7	8	9
Genotype	B	B	C	C	B	C	B	C	B
**Treatment**	TDF	FTC-TDF	TDF	FTC-TDF	TDF	TDF	FTC-TDF	FTC-TDF	FTC-TDF
**Baseline**	9.74E+08	6.99E+08	4.06E+09	9.35E+08	1.74E+09	2.60E+09	1.95E+09	2.81E+09	5.20E+08
**Week 4**	5.82E+05	1.51E+06	4.07E+06	3.21E+06	8.12E+05	2.28E+06	7.73E+07	3.46E+06	1.04E+06
**Week 8**	7.06E+04	8.62E+04	1.56E+06	4.49E+05	2.54E+05	6.72E+05	1.51E+07	9.46E+05	2.67E+05
**Week 16**	1.37E+04	2503	3.12E+05	3.78E+04	5.01E+04	1.66E+05	3.19E+06	9.13E+04	4.55E+04
**Week 24**	918	482	3.07E+04	2436	1.05E+04	8624	5.71E+05	7616	5936
**Week 32**	588	560	9128	734	2072	2660	1.02E+05	1182	946
**Week 40**	1081	381	3662	554	1109	588	2.72E+04	773	470
**Week 48**	1422	381	6888	213	599	862	7504	510	162
**Week 60**	745	162	1053	162	274	291	269	454	162
**Week 72**	190	162	2352	162	162	381	162	342	162
**Week 84**	174	162	1198	162	235	1070	162	174	162
**Week 96**	185	162	566	162	599	409	162	162	162
**Week 112**	207	162	969	162	672	750	162	162	162
**Week 128**	162	162	655	162	162	454	162	162	162
**Week 144**	162	162	750	162	162	218	162	162	162
**Week 160**	162	162	2190	162	162	308	162	162	162
**Week 176**	162	162	2442	162	162	162	162	162	162
**Week 192**	2391	162	734	162	162	162	162	162	162
**Week 208**	162	162	258	162	162	162	162	162	162

None of these patients achieved HBsAg loss or seroconversion during treatment. Serum HBV DNA levels were measured by a COBAS TaqMan assay: the LLOQ of the assay is 162 copies/mL and the LLOD of the assay is 58 copies/mL. Samples with HBV DNA levels <LLOQ but >LLOD were defined as HBV DNA target detected (TD). Of the nine patients selected, only one failed to achieve serum HBV DNA <LLOQ during treatment. All animal studies were performed by PhoenixBio Co. Ltd. (Japan). Unless otherwise specified, patient serum samples were thawed once, aliquoted to limit freeze thaw cycles, and then kept at -80°C. Patient sera were kept frozen until the day of the study and thawed at 37°C.

### Study oversight

All patients provided informed consent [12]. The study was approved by the Institutional Review Board at participating sites and conducted in compliance with the Declaration of Helsinki, Good Clinical Practice guidelines, and local regulatory requirements. The study was designed and conducted by the sponsor in collaboration with the principal investigators. The sponsor collected the data and monitored the study conduct. The investigators, participating institutions, and sponsor agreed to maintain confidentiality of the data.

### In vivo mouse studies

Male uPA/SCID mice between 12–18 weeks of age with humanized liver (cDNA-uPA^wild/+^/SCID [cDNA-uPA^wild/+^: B6;129SvEv-Plau, SCID:C.B-17/Icr-scid /scid Jcl) were produced as previously described by PhoenixBio, Co. Ltd. (Japan) [24]. Briefly, frozen human hepatocytes (donor BD195, Corning Incorporated, Tewksbury, MA, USA) were thawed and transplanted into 2- to 4-week-old uPA/SCID mice by splenic injection. Mice were selected for studies if their liver reconstitution levels had an estimated replacement index greater than 70% based on the blood concentration of human albumin (>8.5mg/mL) one week prior to study initiation. Mice were then randomized into study groups. General health observations including weight were monitored weekly. All animal protocols were performed in accordance with the Guide for the Care and Use of Laboratory Animals and approved by the Animal Welfare Committee of Phoenix Bio Co., Ltd. All mice were housed individually and maintained in accordance with the Animal Ethics Committee of PhoenixBio (resolution #1856, 1916, 2104, 1943, 2102, and 2103).

Human CHB patient serum was administered to the mice via intravenous tail vein injection using disposable 1.0 mL syringes with permanently attached needles. For the titration study, patient sera were diluted in sera from uPA/SCID mice that had not been transplanted with human hepatocytes. The desired target concentration was validated by quantitative real-time PCR with at least 5 technical replicates (see below). Blood was collected under isoflurane (ISOFLURANE Inhalation Solution [Pfizer], Mylan, Osaka, Japan) anesthesia via the retro-orbital plexus/sinus using Intramedic™ Polyethylene Tubing (Becton Dickinson and Compound, NJ, USA) at various timepoints post-infection in each animal. For blood collection at terminal sacrifice, a minimum of 400 μL was collected by cardiac puncture and exsanguination. Of the blood collected, 2 μL was reserved for human albumin measurement. Remaining blood samples were left at room temperature for at least 5 minutes to coagulate and then centrifuged at 13200 x *g* at 4°C for 3 minutes to obtain serum.

### Quantification of human serum Albumin

Blood obtained was diluted in saline and albumin concentration measured by the clinical chemistry analyzer (BioMajesty™ Series JCA-BM6050, JEOL Ltd., Tokyo, Japan) using the latex agglutination immunonephelometry test (LX Reagent “Eiken” Alb II; Eiken Chemical Co., Ltd., Tokyo, Japan). Levels of human serum albumin were measured shortly after inoculation and then at weekly intervals.

### Extraction and quantification of serum HBV DNA

DNA was extracted from 10 μL of serum in two technical replicates (5 μL x 2) by the Smitest Ex-R&D Nucleic Acid Extraction Kit (Medical & Biological Laboratories Co, Ltd, Nagoya, Japan) and dissolved in 20 μL of nuclease-free water (Life Technologies Japan Ltd., Tokyo, Japan). HBV DNA extracted from the serum of a GTC HBV-infected uPA/SCID mouse was used as the HBV DNA standard. HBV DNA copy number in the standard were determined in reference to a synthetic HBV DNA template. The range of standards was 4 x 10^4^ to 2 x 10^9^ copies/mL. The LLOQ of the assay was 4 x 10^4^ copies/mL. The lower LLOD was 8 x 10^2^ copies/mL. Real-time quantitative PCR was performed using the TaqMan Fast Advanced Master Mix (Applied Biosystems, Thermo Fisher Scientific Inc.) and ABI Prism 7500 sequence detector system (Applied Biosystems) using a forward probe (5’—CACATCAGGATTCCTAGGACC—3’), reverse probe (5’–AGGTTGGTGAGTGATTGGAG– 3’) and TaqMan probe (5’ - 6-FAM-CAGAGTCTAGACTCGTGGTGGACTTC-TAMRA– 3’. The initial activation of uracil-N-glycosylase at 50°C for 2 minutes was followed by the polymerase activation at 95°C for 30 seconds. Subsequent PCR amplification consisted of 53 cycles of denaturation at 95°C for 3 seconds and annealing and extension at 60°C for 32 seconds per cycle. The Ct values generated were used to determine HBV DNA copies/mL based on the DNA standard.

### Serum HBV antigen quantification

Serum was sampled biweekly for HBsAg and HBeAg quantification. Serum samples were diluted 120-fold before analysis and measured in duplicate. Serum HBsAg concentration was determined by SRL, Inc. (Tokyo, Japan) using a Chemiluminescent Enzyme Immuno Assay (CLEIA) developed by Fujirebio (LUMIPULSE HBsAg-HQ, LUMIPULSE® Presto II). The detection range of the assay (after dilution) was between 6 x 10^−1^ and 1.2 x 10^10^ IU/mL. Serum HBeAg concentration was also determined by SRL, Inc. using a CLEIA developed by Fujirebio (LUMIPULSE HBeAg, LUMIPULSE® PrestoⅡ). The detection range of this assay (after dilution) was between 1.2 x 10^1^ and 1.9 x 10^5^ C.O.I. Cut-off index (COI) is based on the ratio of assay signal to cut-off signal (also abbreviated s/co).

## Results and discussion

**Fig 1:**
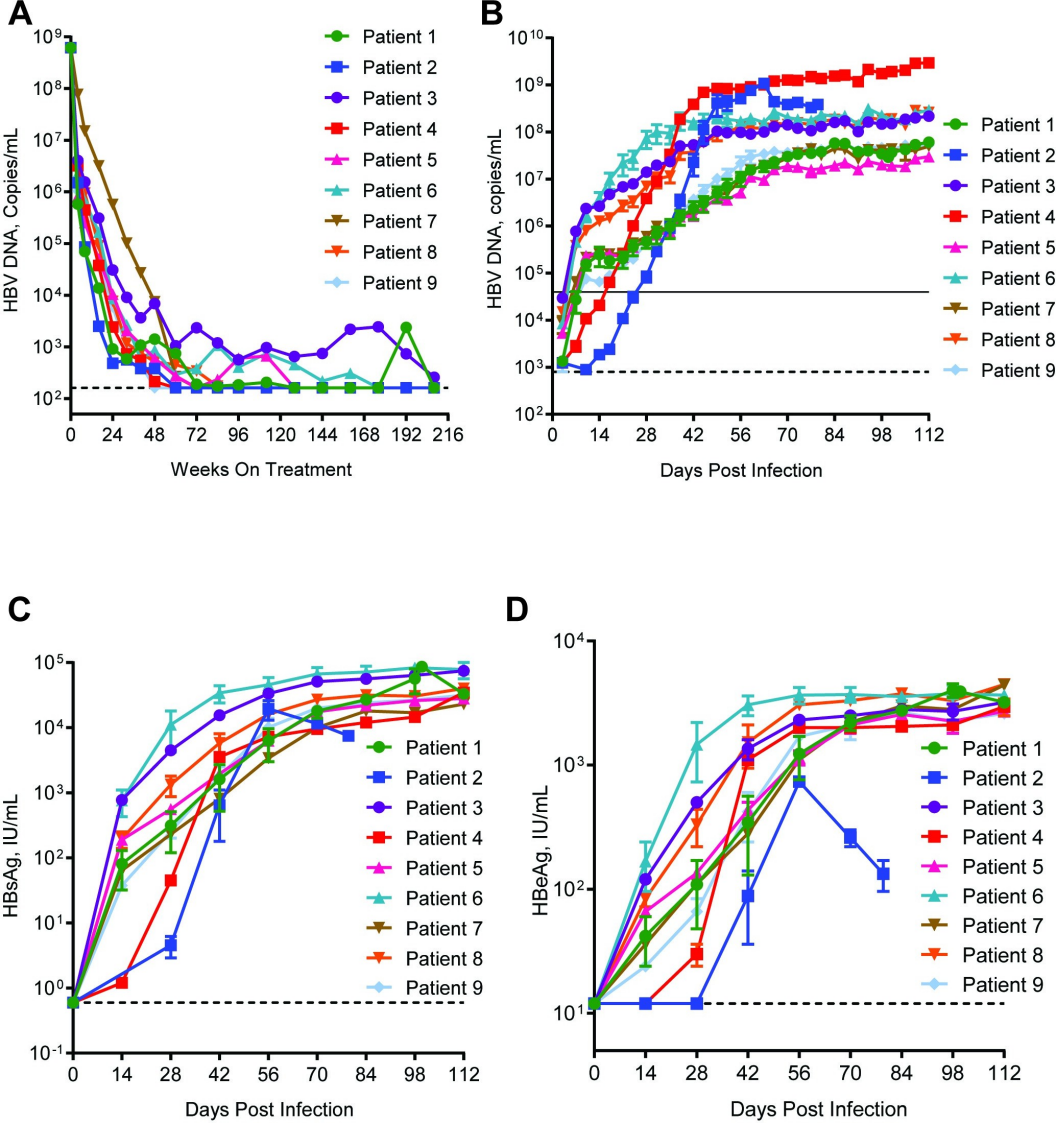
Viral kinetics of 9 patients on NA treatment and when inoculated in the uPA-SCID mouse model. a, Viral kinetics of the patients selected for this study following administration of TDF or TDF/FTC. HBV DNA was measured by Roche TaqMan COBAS at the indicated timepoints and reported as IU/mL and converted to copies/mL (5.6 conversion factor). Each line represents an individual patient. The dashed line represents the lower LLOQ of the assay, 162 copies/mL. b, Mice (n = 2 per isolate) were inoculated with 0.1 mL of baseline serum samples from patients chronically infected with HBV genotype B (open symbols) or C (closed symbols). HBV DNA was measured by qRT-PCR and reported as copies/mL. The dashed line represents the LLOQ (4 x 10^4^ copies/mL) and the solid line represents the LLOD (8 x 10^2^ copies/mL). c, HBsAg was measured by CLEIA (LUMIPULSE HBsAg-HQ) and reported as IU/mL. The dashed line represents the LLOQ (0.6 IU/mL). d, HBeAg was measured by CLEIA (LUMIPULSE HBeAg) and reported as IU/mL. The dashed line represents the LLOQ (12 IU/mL).

Human sera from CHB patients who became suppressed during treatment were available from Gilead study GS-US-203-0101 [12]. To confirm the infectivity of these patient isolates in a mouse model of HBV infection, baseline patient sera were evaluated in the uPA/SCID mouse model. In this model, SCID mice harboring the urokinase plasminogen activator gene and lacking an intact mouse liver are xenotransplanted with human hepatocytes. Mice reconstituted with human hepatocytes can be infected with HBV and the resulting infection resembles chronic infection in humans. High levels of HBV DNA and antigens can be detected in the serum, cccDNA can be isolated from hepatocytes, and mice do not resolve the infection [25,26], due to lack of acquired immune responses [24]. To determine if patient sera can infect chimeric mice, we identified a set of CHB patients (n = 9) infected with HBV genotype B and C which had high viral loads prior to treatment (mean viral load of 1.5 x 10^9^ copies/mL, range from 5.2 x 10^8^ to 4.1 x 10^9^) (Fig 1A, Table 1).

**Fig 2:**
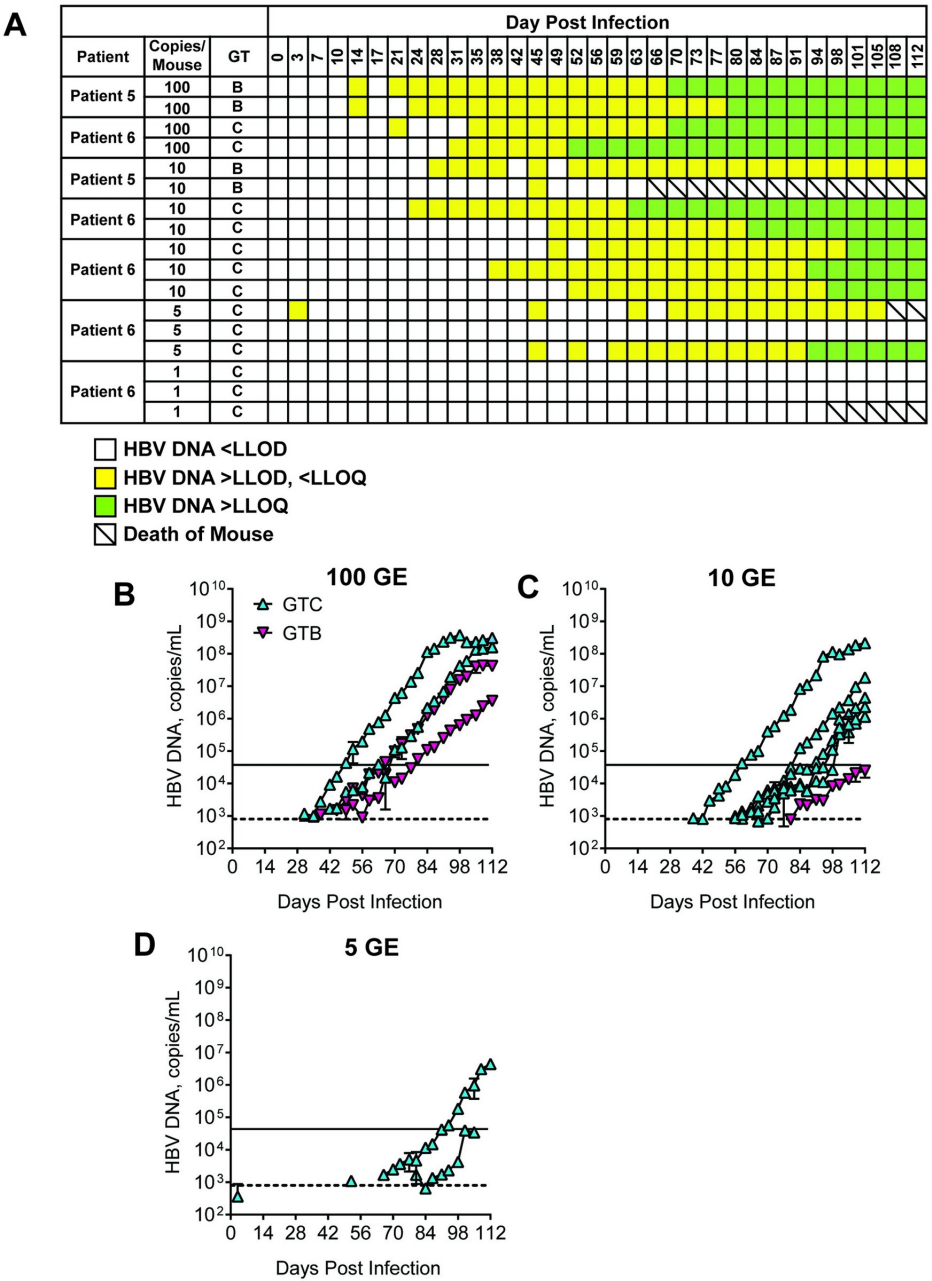
Establishment of the lower LLOD in human liver chimeric mice. Mice were infected with 0.1 mL of sera from patients chronically infected with HBV diluted to contain a total of 100 copies (n = 4), 10 copies (n = 7), and 5 copies (n = 3). a, Summary of PCR results over time. Blank squares indicate HBV DNA values were <LLOD. Yellow squares indicate HBV DNA values were >LLOD but <LLOQ. Green squares indicate PCR was positive and HBV DNA values were >LLOQ. b-d Mean infectivity of mice (n = 2) infected with of 100, 10, and 5 copies of HBV DNA in the uPA-SCID model. HBV DNA was measured by qRT-PCR and reported as copies/mL. Mice infected with genotype B and C isolates are blue and purple, respectively. The dashed line represents the LLOQ (4 x 10^4^ copies/mL) and the solid line represents the LLOD (8 x 10^2^ copies/mL).

**Fig 3:**
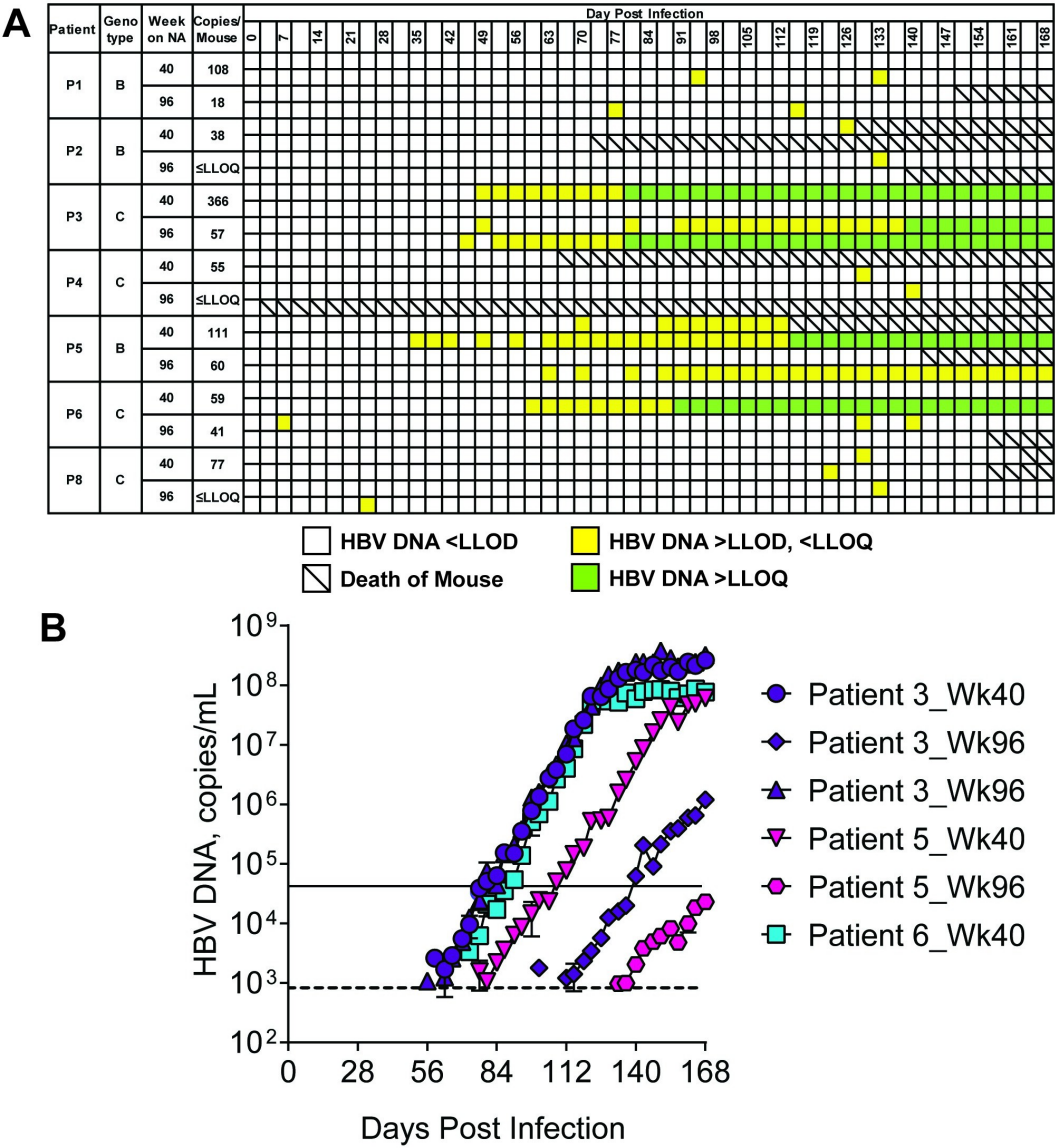
Patient serum near the LLOQ are infectious. Human liver chimeric mice were inoculated with 0.1 mL of sera from CHB patients from a timepoint where the HBV DNA levels were at or below the LLOQ but still TD. a, Summary of PCR results over time. Blank squares indicate HBV DNA values were <LLOD. Yellow squares indicate HBV DNA values were >LLOD but <LLOQ. Green squares indicate HBV DNA values were >LLOQ. b-d, Infectivity of a single inoculation of patient serum into mice (n = 2). HBV DNA was measured by qRT-PCR and reported as copies/mL. The dashed line represents the LLOQ (4 x 10^4^ copies/mL) and the solid line represents the LLOD (8 x 10^2^ copies/mL).

At week 208 all patients were positive for HBeAg and HBsAg and were virally suppressed (≤162 copies/mL) but were classified as having detectable levels of HBV DNA **(Table 1)**. Baseline sera from each patient (n = 9) were inoculated by tail vein injection into the mice (n = 2 mice/patient serum sample) and monitored for the presence of infection. Mouse sera were analyzed biweekly for HBV DNA by quantitative PCR and for HBsAg and HBeAg by ELISA. High levels of HBV DNA, HBsAg, and HBeAg were achieved between 40–60 days post-infection with all patient sera and continued to increase until mice were sacrificed at day 112 post-infection (Fig 1B–1D). Therefore, sera from these CHB patients with high viral titers are able to establish HBV infections in this chimeric mouse model, confirming previous studies with patient sera [21,22]. To determine the level of sensitivity to infection of the mouse model, baseline sera from two patients (one genotype B, one genotype C) were serially diluted such that mice were injected with sera containing 100 (GTB, n = 2; GTC, n = 2), 10 (GTB, n = 2; GTC, n = 5), 5 (GTC, n = 3) or 1 (GTC, n = 3) GE (Fig 2). As before, mice were evaluated for serum markers of HBV infection for 112 days. Mice were considered PCR-positive when HBV DNA levels were detectable but below the LLOQ (Fig 3A, yellow squares) and viremic when serum HBV DNA levels surpassed the LLOQ of the assay (Fig 2A, green squares).

Our findings show that human sera diluted to contain 100 (4/4 mice, 100%), 10 (6/7, 85%), and 5 (2/3, 67%) GE of HBV (Fig 2) became viremic in this mouse model. Correspondingly, sera containing 1 GE failed to establish detectable infection (0/3 mice, 0%), most likely the effect of distribution of very low amounts of virus within a large volume. Mice injected with a higher viral inoculum became viremic faster, with the median time to viremia being 64.5, 94.5, and 96 days for 100 GE, 10 GE, and 5 GE, respectively. Since no mice became viremic after inoculation with 1 GE in these studies, we can conclude that as low inoculum as 5 GE is sufficient for establishing infection in the chimeric mouse model.

Chronic NA treatment is rarely curative in CHB patients and a lack of complete viral suppression is one potential explanation. To test the hypothesis that long-term NA-treated virally suppressed patients still have low levels of circulating infectious virus, we injected mice with 0.1 mL sera from CHB patients (n = 7) after ≥40 weeks of NA treatment (Fig 3A, Table 1).

Mice (n = 2 per patient) were inoculated with serum from each patient taken at either week 40 or week 96 of treatment. HBV DNA for each patient at each time point are listed as copies per milliliter in Table 1 and copies per mouse inoculation in Fig 3A. Mouse sera were analyzed biweekly for HBV DNA by quantitative PCR and for HBsAg and HBeAg by ELISA. Six of the 28 mice inoculated with patient sera became viremic (Fig 3B). Mice that became viremic received serum from timepoints at which patient sera was near–but still above–the LLOQ (patient 3, week 40 and week 96; patient 5, week 40 and week 96; and patient 6, week 40). However, none of the mice that received patient sera whose HBV DNA levels were TD but below the LLOQ, became viremic (Fig 3).

**Fig 4:**
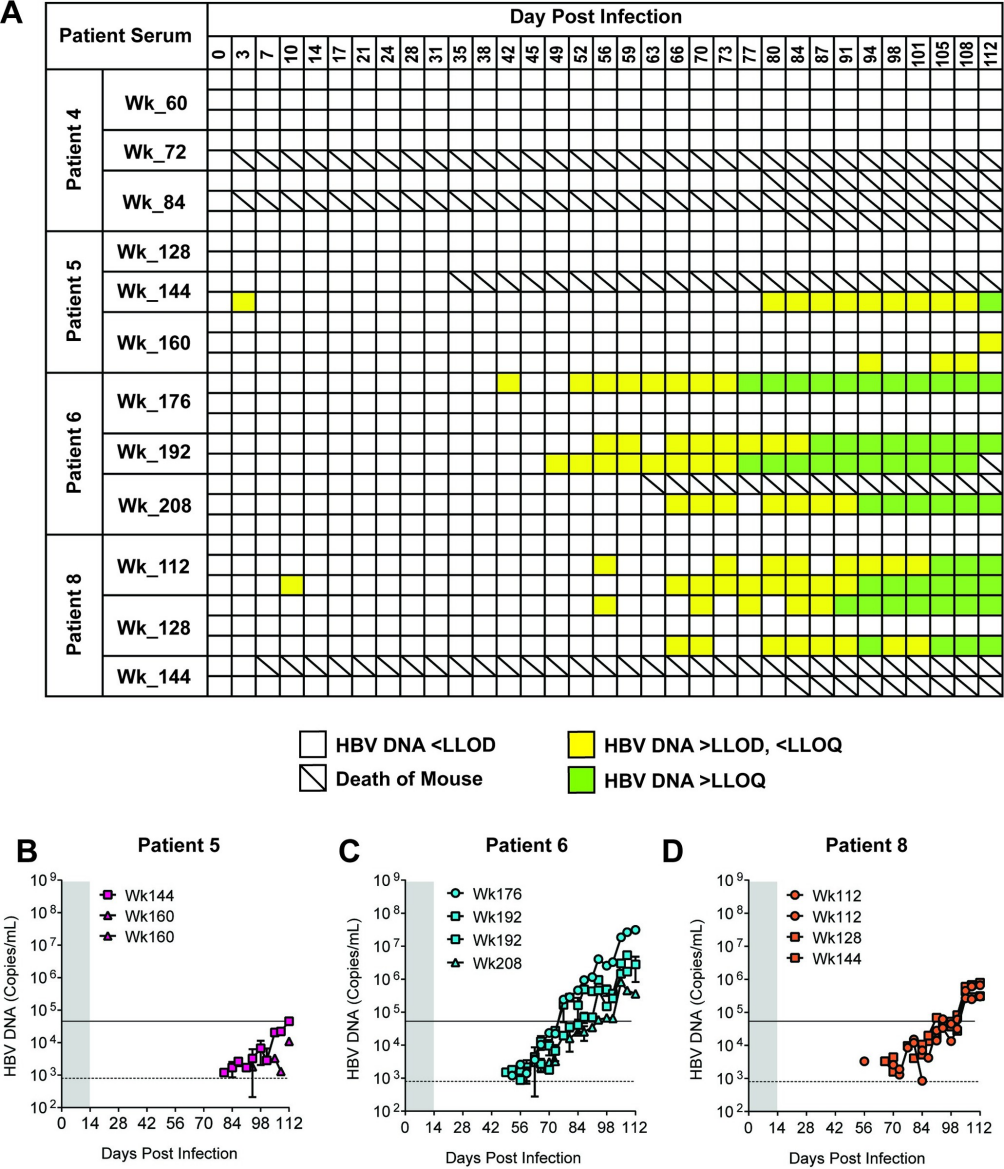
Patient serum samples below the LLOQ contain infectious virus. Human liver chimeric mice (n = 31) were inoculated with sera from CHB patients with HBV DNA levels <LLOQ but TD. a, Summary of PCR results over time. Blank squares indicate HBV DNA values were <LLOD. Yellow squares indicate HBV DNA values were >LLOD but <LLOQ. Green squares indicate HBV DNA values were >LLOQ. b-d, Infectivity of sera from NA-treated patients suppressed to below the LLOQ. HBV DNA was measured by qRT-PCR and reported as copies/mL. The dashed line represents the LLOQ (4 x 10^4^ copies/mL) and the solid line represents the LLOD (8 x 10^2^ copies/mL). Shaded gray area represents the two-week period over which mice were administered a total of 5 consecutive serum inoculations.

Each 100 μL injection of sera from a patient whose HBV DNA level was TD, but below LLOQ (≤162 copies/mL), nominally contains ≤16.2 copies. Given the small sample volume, the stochastic variation in viral inoculum size may have resulted in many of the patient serum injections containing fewer than 5 GE HBV which is below the sensitivity limit of the mouse model. Accordingly, mice (total n = 31) were inoculated with five serial inoculations of patient serum taken from the same patient and same timepoint over a two-week period. Four patients were chosen (Patient 4, 5, 6 and 8) whose serum HBV DNA levels were ≤ LLOQ on treatment (Table 1, Fig 4A).

Mice became viremic after receiving serial inoculations from patient 5, 6, and 8, but not patient 4. In contrast to the mice receiving single inoculations, a subset of mice that received serial inoculations of sera became viremic (n = 9/31; 29%) at a median time of 66 days post-infection (Fig 4). These results indicate that patients considered suppressed on NA therapy can have circulating infectious virus.

NAs have been shown to be safe and effective in reducing HBV viral load, improving inflammation and fibrosis, and reducing both the liver failure and the rate of HCC for patients with HBV. However, even after years of NA treatment the majority of CHB patients still have detectable HBV DNA in their serum [27]. The nature of this HBV DNA in suppressed patients is hypothesized to reflect infectious virus, but this has never been definitively shown. Here, we show that sera from patients on long-term NA therapy are capable of establishing infection in a chimeric mouse model of HBV. Furthermore, serum from patients whose HBV DNA is suppressed below the LLOQ (but still TD) is infectious, indicating that patients continue to actively replicate HBV even on long-term suppressive NA therapy.

For this study, sera were selected from patients enrolled in the GS-US-203-0101 study, a phase 3 clinical trial evaluating long-term treatment of TDF alone or TDF in combination with FTC. All of the patients were infected with either HBV genotype B or genotype C and became HBV DNA suppressed (≤LLOQ or ≤162 copies/mL) at some point within the 208 weeks of treatment (Fig 1A). An immunodeficient mouse model (uPA-SCID) was used in this study to evaluate the infectivity of patient isolates. The model was able to support infection from baseline patient serum (Fig 1B), the kinetics of infection with these patient isolates mirroring what has been observed for mouse-adapted strains [22]. Based on the data with the current small sample size, we observed that mice infected with genotype C patient virus achieved higher maximal viral loads than genotype B. This is most apparent in Fig 2 where all (n = 4/4) GTC viruses achieved a peak viral load at 1 x 10^8^ copies/mL or greater whereas GTB (n = 4/5) viruses achieved peak viral load at 5 x 10^7^ copies/mL. Furthermore, when both viruses and were diluted to 100 GE per inoculum, infection with 100 GE of GTC viruses resulted in mice becoming viremic earlier than mice infected with GTB isolates (Fig 3B). It is not known if the genotype differences in peak viremia reflect differences in infectivity and/or production of mature virus, However, levels of HBeAg and HBsAg achieved with both genotypes were equivalent favoring the latter scenario (Fig 1C and 1D).

We performed a dilution series study to ascertain the lower limit of infectivity of the uPA-SCID mouse model with respect to human serum and established that the lower limit of infectivity of the model to be > 1 and ≤ 5 GE (Fig 2). Two viral isolates, one each from genotype B and C, were used in this study, however, the actual lower limit of sensitivity could vary among clinical isolates. To test the hypothesis that long-term NA-treated virally suppressed patients still have low levels of circulating infectious virus, mice were inoculated with a single dose of serum. The only mice that had viremia were those dosed with serum > LLOQ (Fig 3). However, there were a few caveats to this study. First, the probability that a sample contains infectious virus in each volume decreases as the GE lowers. Thus, due to the sensitivity limit of the model, serum might still contain infectious virus, even if the inoculum did not result in viremia. Second, while the study lasted 168 days, it is possible that more mice would have become viremic had the study continued. To this point, at the conclusion of the study, several mice were PCR positive but did not have viral loads above the LLOQ (Fig 3A).

The critical result from this study was that serum with HBV DNA levels ≤ LLOQ (but still TD) taken from CHB patients after ≥112 weeks of NA therapy gave rise to viremia in mice (Fig 4). In this instance, an individual mouse received multiple inoculations of the same sera. In some cases, serum from patients suppressed on therapy for as long 48 weeks (median 34 weeks, range 24–48 weeks) was able to cause viremia in mice, indicating that those patients on long-term therapy still contained infectious virus. However, there were patient sera that did not give rise to viremia in mice, even following multiple inoculations. As with all the studies, lack of viremia does not necessarily indicate the complete absence of infectious virus. Extending the duration of the study and/or increasing the number of injections could enhance the positivity rate. At the time of the study, we did not have access to serum from patients who achieved viral suppression and had undetectable levels of HBV DNA (≤162 copies per mL and TND). Additional studies will be necessary to determine if such samples contain infectious virus.

This is the first study to demonstrate the presence of infectious virus in the serum of patients who are suppressed on NA therapy with DNA levels ≤LLOQ and TD. The observation that the residual viral DNA represents infectious virus has implications for the development of curative treatments for CHB. Consistent with our findings, patients who are virally suppressed on NA therapy maintain levels of serum HBV RNA and detectable intrahepatic cccDNA [28,29]. We assume this cccDNA in NA-treated patients is transcriptionally active, given the presence of HBV RNA and the presence of other viral markers coming from cccDNA (HBcrAg and HBeAg) [30]. Low-level viremia in NA-treated patients may continue to serve as a reservoir of new infections, leading to the *de novo* formation of cccDNA and maintenance of chronic HBV infection in the liver albeit at a more reduced rate than in untreated patients. Moreover, as NAs do not completely block viral replication, HBV integration events are also likely to continue. Therefore, the residual viremia may also explain the continued risk, although low, for the development of long-term HBV-related complications, such as HCC, in virally suppressed patients. In support of this fact, studies have demonstrated that patients on NA therapy that do achieve functional cure have a reduction in HCC risk compared to those that merely remain virally suppressed [31,32].

Finally, these results highlight that the full suppression of infectious HBV replication in CHB patients would require combination of NAs with additional orthogonal agents (e.g., capsid modulators, siRNA). Theoretically, if it is possible to achieve complete viral suppression with a combination of such agents, the natural turnover of hepatocytes should eventually eliminate all cccDNA containing cells. Thus, the duration of treatment with such agents would have to exceed the time it takes to turn over all infected cells. Recent reports determined the half-life of cccDNA to be approximately 12–24 weeks [33]. Others have reported the cccDNA half-life can range from 2 weeks to 33 weeks, depending on HBeAg status [34]. Even if the half-life of cccDNA or infected cells is longer than predicted, completely stopping viral replication and preventing the reinfection of new cells (or intracellular replenishment of cccDNA in infected cells) is a potentially important component of curative strategies along with establishment of an effective host immune response [35]. Toward this end, there are many active clinical studies combining NAs with a variety of additional agents including those that act by antiviral and immune modulatory mechanisms; results from these trials will yield critical insights to continue refinement of strategies for successfully achieving HBV cure.

## Supporting information

S1 Table Raw data for Figs 1–4. (XLSX)
